# Influence of Dietary Inclusion of Exhausted Bergamot By-Product in Pigs on Animal Performance, Fatty Acid Profile and Oxidative Stability of Meat and Meat Products

**DOI:** 10.3390/ani12060757

**Published:** 2022-03-17

**Authors:** Manuel Scerra, Rosa Rao, Francesco Foti, Pasquale Caparra, Caterina Cilione, Antonio Natalello, Luisa Biondi, Marco Sebastiano Bella, Luigi Chies

**Affiliations:** 1 Dipartimento di Agraria, Produzioni Animali, University of Reggio Calabria, Via dell’Università, 25, 89124 Reggio Calabria, Italy; manuel.scerra@unirc.it (M.S.); rosa.rao@unirc.it (R.R.); francesco.foti@unirc.it (F.F.); pasquale.caparra@unirc.it (P.C.); caterina.cilione@unirc.it (C.C.); lchies@unirc.it (L.C.); 2 Dipartimento di Agricoltura, Alimentazione e Ambiente (Di3A), University of Catania, Via Valdisavoia 5, 95123 Catania, Italy; luisa.biondi@unict.it (L.B.); marco.bella@unict.it (M.S.B.)

**Keywords:** bergamot pulp, pigs, meat quality

## Abstract

**Simple Summary:**

The pharmaceutical industry is increasingly interested in the extraction of phenolic compounds from bergamot pulp, which becomes less available for use in animal feeding. On the other hand, the extraction of phenol compounds generates a new bergamot by-product characterized by a low moisture content and a small concentration of phenolic compounds which are residual after extraction. In light of the above, the aim of the present study was to investigate the effect of supplementation of exhausted bergamot by-product in pigs’ performance and on the quality of meat and meat products.

**Abstract:**

An investigation was carried out on the effect in pig diet of supplementation with exhausted bergamot by-product, stemming from pharmaceutical industry after extraction of phenolic compounds, on growth performance and on the quality of meat and meat products. Twenty pigs were assigned to two dietary treatments and fed a conventional concentrate (control) or a concentrate including exhausted bergamot by-product at the level of 15% on a DM basis (EB). No significant differences between dietary treatments were found for final weight (*p* = 0.243), carcass weight (*p* = 0.679), dry matter intake (*p* = 0.321). In EB pork, the proportion of docosapentaenoic acid was significantly increased (*p* < 0.05); it tended to have a greater proportion of *n*-3 PUFA (*p* = 0.09), and the *n*-6/*n*-3 PUFA ratio was lower in EB treatment (*p* = 0.01). In salami from EB pigs fed, the proportion of α-linolenic acid and the total *n*-3 PUFA were higher than in the control group (*p* < 0.001). In salami, the TBARS value was lower after 5 days of storage (*p* < 0.001) in the EB group. Therefore, the present results suggest that the inclusion of exhausted bergamot by-product in pig diet resulted in a qualitative improvement of meat and meat products.

## 1. Introduction

In the last decade, there has been an enormous increase in the interest in the use of agro-industrial by-products as animal feed; consequently, there has been an increase in the prices of conventional raw materials, and this has forced farmers to modify the feeding systems of the animals accordingly. Therefore, the use of local feed resources appears to be able to offer economic advantages through the reduction of conventional feed and environmental costs in terms of reducing the impact that would derive from the need to dispose of many of these biomasses [1].

Fruit of the genus *Citrus* are widespread in the Mediterranean area. The bergamot (*Citrus Bergamia* Risso) is a hybrid fruit derived from bitter orange and lemon. It is abundantly produced in the south of Italy to obtain juice and a valuable essential oil. However, a significant amount of waste, which is generally named as bergamot pulp, originates from the processing of bergamot fruit. Similar to other citrus pulps (e.g., orange and lemon pulp), bergamot pulp could be used in ruminant’s nutrition, due to its favorable nutritional composition, which makes it, especially in dehydrated form, a valid substitute for cereals in the formulation of concentrates, without problems on the production efficiency of animals [2]. Furthermore, fibrous feedstuffs such as citrus pulp—which, in the past, were not considered for monogastrics [3]—have been reevaluated for use in pig diets in the last few years [4].

Some studies that investigated the chemical composition of bergamot pulp have reported the presence of some flavonoids, such as naringin and neoesperidin [5,6]. Flavonoids are substances that show pharmacological and biological properties: anti-inflammatory, antimicrobial and antiviral activity; effects on capillary fragility; inhibition of platelet aggregation (antithrombotic effect); and antitumor activity [7,8,9,10]. Moreover, it is precisely naringin that is associated with probable protective actions on pre–neoplastic lesions [11]. Furthermore, a particular interest in the pharmaceutical–nutritional field is related to their antioxidant capacities.

The use of this citrus by-product in animal feeding may therefore induce some possible antioxidant effects in products of animal origin, including meat [12]. In accordance with this hypothesis, a recent study [13] has demonstrated that feeding ensiled bergamot pulp to pigs had a positive effect on meat products’ oxidative stability, a result probably also linked to the good level of phenolic compounds present in the tested by-product. However, the pharmaceutical industry is increasingly interested in the extraction of phenolic compounds from bergamot pulp, which becomes less available for use for animal feeding. On the other hand, the extraction of phenol compounds generates a new bergamot by-product characterized by a low moisture content and a small concentration of phenolic compounds which are residual after extraction.

To our knowledge, no studies tested the dietary inclusion of the latter bergamot by-products from pharmaceutical industry on performance and on the quality of meat and meat products in pigs. Therefore, the objective of this study was to estimate the effect of including exhausted bergamot pulp in pig diet on animal performances, fatty acid composition and the oxidative stability of meat and meat products.

## 2. Materials and Methods

### 2.1. Dried Bergamot Pulp

Dried bergamot pulp was provided from a pharmaceutical company after the extraction of phenolic compounds (H&AD—Herbal & Antioxidant Derivatives Company, Bianco, RC, Italy). The by-product was transported at 4 °C to the laboratory of the University of Reggio Calabria and stored in the dark at the same temperature.

The extraction of these compounds was carried out by using the bergamot fruit without its external cuticle. Columns containing polystyrene adsorbent resin were used to absorb polyphenols, which were then eluted and recovered, following further steps. After the extraction, the residual phenol compounds in dried bergamot pulp were 6.39 g TAe/kg dry matter.

### 2.2. Experimental Design

The experiment was conducted with pigs in collaboration with a farm oriented on pig production, and the experimental protocol was approved by the Animal Welfare Committee (O.P.B.A) of the University of Reggio Calabria (prot. No. 16331). Twenty castrated Apulo-Calabrese pigs in the finishing phase were assigned to two dietary treatments and fed with a conventional concentrate (control treatment) or with a concentrate including exhausted bergamot by-product at the level of 15% dry matter (EB). The diets were formulated to be isonitrogenous and isoenergetic (Table 1). Control treatment: barley 30%, maize 30%, oat 16%, soybean meal 7%, faba bean 14% and vitamin mineral premix 3%. EB treatment: barley 23%, maize 23%, oat 12%, soybean meal 10%, faba bean 14%, exhausted bergamot pulp 15% and vitamin mineral premix 3%. Water was continuously available.

The pigs were weighed (95.2 ± 3.3 kg initial body weight and 16 ± 0.5 months of age), assigned to two experimental treatments consisting of 10 animals each and allocated to individual pens (4 m^2^) fitted with a metal trough and nipple water dispenser. After a period of adaptation to the experimental diet of 10 days, the barrows were fed ad libitum for 120 days.

All the pigs were fed twice daily, at 8:00 a.m. and 5:00 p.m. For each pig, individual feed intakes were measured daily, and body weight was measured every two weeks.

### 2.3. Slaughter Procedure and Sampling

At the end of the trial, all animals were slaughtered on the same day in a commercial abattoir, according to the European Union welfare guidelines (council regulation (EC) No. 1099/2009). Thereafter, the carcass weight was recorded, and the muscle longissimus thoracis et lumborum (LTL) and the corresponding subcutaneous fat were removed from each carcass. One part of LTL muscle and of subcutaneous fat were immediately refrigerated and transported to the laboratory for the analysis. The remaining parts of the samples were devoted to producing salami from each animal. Therefore, 20 salamis were prepared (10 per treatment). In a local company, all the salamis were produced with the same technology, on the same day. They used the back fat and meat of each pig to prepare individual salami, using meat (90%, *w*/*w*), back fat (10%, *w*/*w*) and 25 g/kg of salt. After mixed, the paste obtained was stuffed, tied, hung and placed in a fermentation chamber, where, for the first 24 h, the salamis were kept at a temperature of 20 ± 2 °C, with a relative humidity of 75–80%, to gradually reduce it to 10–12 °C within five days, while the relative humidity was gradually increased to 80 ± 5%.

After 30 days of maturation, the salamis were vacuum-packed (C308, Raja) and stored at −20 °C for subsequent laboratory analysis.

### 2.4. Feedstuff Analysis and Meat Proximate Analysis

Feed samples were analyzed for moisture (method 930.15), ash (method 942.05), crude protein (method 984.13, Kjeldahl method) and crude fat (method 920.39) according to Association of Official Analytical Chemists procedures [14]. Feed samples were analyzed for neutral detergent fiber (NDF) [15]. Total phenolic compounds were evaluated according to the procedure described by Makkar et al. [16], using aqueous acetone (70% *v*/*v*), as extraction solution and Folin–Ciocâlteu reagent. The total phenolic compounds were expressed as grams of tannic acid equivalents per kg of dry matter. Lipids, for the analysis of fatty acid, were determined by using the method of Gray et al. [17]. Tocopherols and retinol were extracted from 50 mg of exhausted bergamot by-product and 200 mg of concentrates, as reported by Rufino-Moya et al. [18], using 3 mL of methanol:acetone:petroleum ether (1:1:1, *v*:*v*:*v*) with BHT (0.01% *w*/*v*). Then, the supernatant was collected, evaporated and 1 mL of methanol was added. Dissolved sample was then filtered by using a 0.22 µm PTFE filter into a 2 mL vial. Liposoluble vitamins were analyzed by using an ultra-high performance liquid chromatography (UHPLC; Nexera, Shimadzu Corporation, Kyoto, Japan), as later detailed for the determination of meat tocopherols.

In samples of longissimus thoracis et lumborum muscle moisture (method 950.46), ash (method 920.153), crude protein (method 984.13) and crude fat (method 991.36) were analyzed according to Association of Official Analytical Chemists methods [14], after 24 h of thawing at 4 °C.

### 2.5. Fatty Acid Analysis and Antioxidant Vitamins Analysis in Meat and Meat Products

Fatty acid composition was analyzed on total lipids extracted according to the procedure of Folch et al. [19].

Briefly, 5 g of homogenized sample was blended with a mixture of chloroform and methanol (2:1, *v*/*v*) mixed with saline solution (0.88% KCl). Methylation was performed in duplicate according to IUPAC method [20]; the internal standard used was C9:0. The FAME separation was carried out as reported by Scerra et al. [13], using a Varian gas chromatograph (model CP 3900, Varian, Mitchell Drive, Walnut Creek, CA, USA) equipped with a capillary column CP-Sil 88 (100 m × 0.25 mm i.d., film thickness 0.25 μm).

Atherogenic and thrombogenic indexes (AI and TI, respectively) were evaluated by using the formulas reported by Ulbricht and Southgate [21] as follows: AI = (C12:0 + 4 ∗ C14:0 + C16:0)/(MUFA + PUFA *n*-6 + PUFA *n*-3) and TI = (C14:0 + C16:0 + C18:0)/(0.5 MUFA + 0.5 PUFA *n*-6 + 3 PUFA *n*-3 + PUFA *n*-3/PUFA *n*-6). Highly peroxidizable (HP) PUFA was calculated as the sum of PUFA with ≥3.

Vitamin concentrations of muscle and salami were determined as reported by Natalello et al. [22], using an UHPLC system.

### 2.6. Lipid Oxidation and Color Measurements

Oxidative stability over aerobic refrigerated storage was measured in raw and cooked meat and in salami, using 2 cm–thick slices of muscle and salami samples. Shortly, three slices were placed in polystyrene trays, covered with vinyl film and stored in the dark in a refrigerated environment (4 °C), and each slice was used for measuring lipid oxidation at each refrigerated storage time (after 2 h of blooming, 3 and 7 days of storage in raw meat; and after 2 h of blooming, 2 and 5 days of storage in cooked meat and salami [23]). For assessing the extent of oxidative stability in cooked meat, the slices were vacuum-packaged and cooked by bags immersion into a water-bath set at 75 °C (WBH100, MRC, Hagavish 3, Holon, Israel). After 30 min of cooking, the samples were cooled by immersion of the bags into a water/ice bath. The bags were opened, and 1 slice was used immediately for measuring the extent of lipid oxidation (day 0). The remaining slices were placed into polystyrene trays, overwrapped with vinyl film and stored in the dark at 4 °C.

Lipid oxidation was measured by evaluating the 2-thiobarbituric acid reactive substances (TBARS) at the end of each storage time, as reported by Siu and Draper [24], using a Shimadzu double-beam spectrophotometer (model UV—1800; Shimadzu Corporation, Milan, Italy).

Color stability in raw meat was performed by a Minolta CR300 color meter (Minolta Co. Ltd., Osaka, Japan), with illuminant A and 10° standard observer, on the same slices used to asses lipid oxidation, at the end of each storage time (0, 3 and 7 days). The average of three measurements, taken on the slice surface, were used to calculate L* (lightness), a* (redness), b* (yellowness), C (saturation) and H (hue angle) values of the CIE L* a* b* color space.

### 2.7. Statistical Analysis

All the data were analyzed with the statistical software Minitab, version 16 (Minitab Inc, State College, PA, USA). The effect of dietary treatments on animal performance and intramuscular FA composition were analyzed by using one-way ANOVA. Data on color and lipid oxidation in raw meat, cooked meat and salami were analyzed by using a mixed model for repeated measures, while individual animal was included as a random factor. When the *p*-value was ≤0.05, means were separated through Tukey’s adjustment for multiple comparisons.

## 3. Results

### 3.1. Animal Performance and Chemical Composition of Meat and Salami

Data on animal performances are reported in Table 2. No significant differences between dietary treatments were found for final weight (*p* = 0.243), carcass weight (*p* = 0.679), dry matter intake (DMI; *p* = 0.321), average daily gain (ADG; *p* = 0.472) and feed-conversion ratio (FCR; *p* = 0.191).

The daily intake of total fatty acids (FA) was greater (*p* < 0.01) for the animals fed the diet supplemented with exhausted bergamot pulp compared to the control diet.

Regarding individual fatty acids, stearic acid intake (*p* = 0.073) and linoleic acid intake (*p* = 0.098) tended to increase in the EB group compared to the control group, and the EB group ingested a higher concentration of α-linolenic acid (*p* = 0.001).

The chemical composition of the meat and salami samples is presented in Table 3. No significant differences between groups were found for moisture, crude protein, ether extract and ash for both (*p* > 0.05).

### 3.2. Fatty Acid Composition of Intramuscular Fat and Antioxidant Vitamins

The concentrations of vitamins E and A in the meat are reported in Table 4. The concentration of vitamin E (VE) was not affected by dietary treatment (*p* > 0.05), while the concentration of retinol (vitamin A) tended to be influenced by the diet, being greater in the group treated with the EB (*p* = 0.078).

The effect of dietary treatment on the fatty acid composition of longissimus thoracis et lumborum is reported in Table 4. No effect was found (*p* > 0.05) for the proportion of saturated fatty acids (SFA), monounsaturated fatty acids (MUFA) and polyunsaturated fatty acids (PUFA). Individual fatty acids were not affected by the dietary treatment (*p* > 0.05), except the proportion of C22:5 *n*-3 (DPA), which was increased by the EB inclusion (*p* = 0.023). Furthermore, the LTL muscle from pigs fed with EB diet tended to have a greater proportion of *n*-3 PUFA (*p* = 0.08); as a consequence, the *n*-6/*n*-3 PUFA ratio was lower in the EB treatment (*p* = 0.01).

As regards the meat concentration of the highly peroxidizable (HP) PUFA, with unsaturation degree ≥ 3, no significant differences were found between the two groups (*p* > 0.05). Nevertheless, the HP-PUFA ÷ Tocopherols ratio tended to increase (*p* = 0.057) in meat when the pigs were fed the EB diet compared with the control diet.

The thrombogenic index tended to be reduced (*p* = 0.064) by feeding pigs with the EB diet compared to the control diet.

### 3.3. Fatty Acid Composition of Back Fat

The fatty acid composition of back fat is reported in Table 5. The EB treatment tended to increase the total PUFA concentration (*p* = 0.072) in back fat compared to the control diet, while no effect was found for the sum of SFA and MUFA (*p* > 0.05).

When evaluating the individual fatty acids, we noted that back fat from pigs fed the EB diet had a greater proportion of α-linolenic acid (*p* < 0.05).

Overall, the proportion of n-3 PUFA in fat tended to increase (*p* = 0.057) by feeding pigs the EB diet compared with the control diet.

### 3.4. Fatty Acid Composition of Salami and Antioxidant Vitamins

The fatty acid composition of salami is reported in Table 6. Salami from pigs fed the EB diet had a smaller proportion of total SFA (*p* < 0.05). In fact, by evaluating the individual fatty acids, it emerged that both myristic acid and palmitic acid presented a lower proportion, tending to significance (C14:0, *p* = 0.098; C16:0, *p* = 0.099), while the stearic acid was significantly lower (C18:0, *p* = 0.036).

No significant effects were found for the proportion of MUFA (*p* > 0.05), while the proportion of polyunsaturated fatty acids (PUFA) in salami from pigs was affected by the dietary treatment (*p* < 0.001), with the highest value found in the salami from pigs fed the EB diet. Salami from pigs fed the EB diet had a greater proportion of α-linolenic acid (*p* < 0.001) and tended to have a greater proportion of linoleic acid (*p* = 0.081) compared to salami from pigs fed the control diet.

No effect was found (*p* > 0.05) for the other long-chain *n*-3 polyunsaturated fatty acids, such as eicosapentaenoic (EPA), docosapentaenoic (DPA) and docosahexaenoic (DHA).

Regarding the *n*-6/*n*-3 ratio and the PUFA/SFA ratio, both were significantly influenced by dietary treatment, (*p* < 0.001 and *p* = 0.004, respectively), presenting a better value in salami from pigs fed with the EB diet.

The thrombogenic and atherogenic indexes were affected by dietary treatment (*p* = 0.001 and *p* < 0.05 respectively), with the lowest values found in salami from pigs fed the EB diet compared with the control group.

No significant effects were found for the levels α-tocopherol, γ-tocopherol and retinol (Table 6) in salami. Supplementing exhausted bergamot by-product in the finishing diet of pigs significantly increased the concentration of the HP-PUFA (*p* = 0.001) in salami. Consequently, the EB treatment also increased (*p* = 0.033) the HP-PUFA ÷ Tocopherols ratio compared to the control group.

### 3.5. Meat and Salami Oxidative Stability and Meat Color

The effect of the dietary treatment and time of refrigerated storage on the oxidative stability parameters measured in raw and cooked meat is reported in Table 7. The dietary treatment influenced some color parameters measured in raw meat. In particular, L* values (*p* = 0.038) and b* values (*p* = 0.085) were higher in the EB diet. Moreover, the time of storage influenced some color parameters measured in meat during storage—in particular b* values (*p* = 0.001) and H* values (*p* = 0.001)—and also tended to influence C* values (*p* = 0.079).

In raw and cooked meat, lipid oxidation (TBARS values) was not affected by the dietary treatment (*p* > 0.05), whereas TBARS values increased during the days of storage (*p* = 0.001) only for cooked meat.

**Figure 1:**
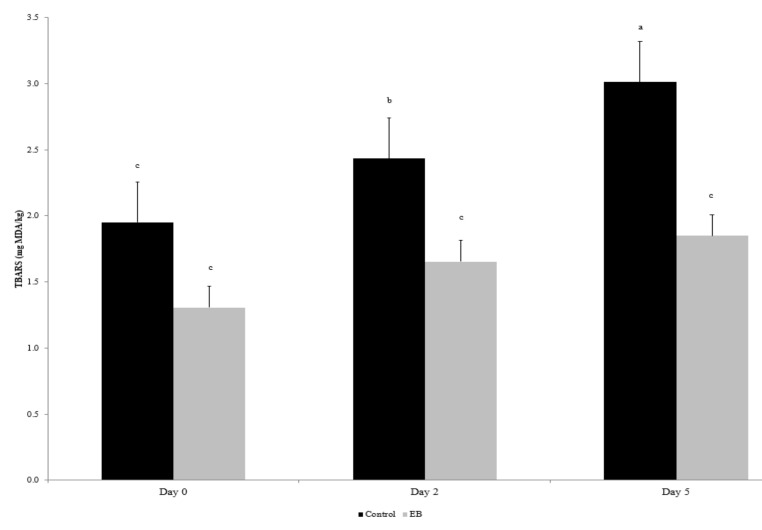
Lipid oxidation (TBARS assay) measured in salami slices over 5 days of aerobic storage at 4 °C. Values are the means and standard error bars. ^a,b,c^ Different letters indicate differences between means (*p* ≤ 0.05).

The lipid oxidation of salami is reported in Figure 1. The dietary treatment significantly influenced (*p* < 0.001) the extent of lipid oxidation in salami; indeed, the TBARS values were lower in the EB diet. Furthermore, the TBARS values increased during aerobic refrigerated storage (*p* < 0.001), and after 5 days, the salami of the EB group showed lower values compared to the salami of the control group (*p* < 0.01).

## 4. Discussion

### 4.1. Growth Performance

To date, the use of by-products in animal nutrition is widely documented. In fact, it is also thanks to the inclusion of by-products in animal feeding that strategy have been pursued to improve environmental and economic sustainability. Ali et al. [25] observed that using by-product, as citrus pulp (dried), in the diets of pigs has the potential to reduce environmental impact of pork production in terms of global warming potential and allows for the use of land for the food crops production intended for human food.

As claimed by Kyriazakis and Emmans [26], an abundant presence of dietary fibre in pig diet can lead to a reduction of feed intake, due to the specific polysaccharides such as pectins, which absorb water and form a gelatinous compound within the intestinal lumen. Other authors [27] showed that the inclusion of 5% and 10% of ensiled citrus pulp in the diet for growing pigs resulted in a reduction of animal growth performance. This negative effect may derive from the reduction of DM feed intake during the first 4 weeks of the experimental trial, but the differences subsequently disappeared.

In the current study, no significant differences were observed in terms of DMI between treatments. Probably, supplementing the bergamot by-product as dried, ground and mixed with the remaining ingredients of the diet affected this result.

### 4.2. Fatty Acid Composition

Apulo-Calabrese, similarly to the other Italian local pig breeds [28], is characterized by reduced growth and carcass performance [29]. Growth is slow, and this was probably the main cause that led breeders to prefer other breeds that are earlier and with a better feed conversion index. Actually, this slow growth, if associated with a balanced diet, could have an effect on the fatty acid profile of the meat and therefore affect the nutritional quality of the food itself. However, in our trial, we also evaluated the fatty acid composition of meat, because Aboagye et al. [29] showed that, when the Apulo-Calabrese pigs are managed in the same rearing conditions as crossbreeds, their muscle fatty acid composition was comparable; therefore, the fatty profile of the meat seems to not be influenced by the genetic type. Conversely, the fatty acid composition of meat can be influenced by the fatty acid composition of the experimental diet. Wood and Enser [30] as early as 1997 asserted that, in all species, meat fatty acid composition can be modulated by the diet, especially more easily in pigs, monogastric animals, where the linoleic, α-linolenic and long-chain PUFA respond quickly to high dietary concentration.

Despite the fact that meat has been and still is criticized because of its undesirable fatty acid profile, due to the high proportion of SFA rather than PUFA, meat is an essential food for the human diet. Meat is a valuable source of high biological value protein, iron, vitamin B_12_ and other B complex vitamins, zinc, selenium and phosphorus. In fact, the elimination of meat from the diet could increase the risk of severe nutritional deficiencies and impair human health and nutritional status [31]. Moreover, pork has a favorable balance between polyunsaturated and saturated fatty acids (PUFA/SFA), and our data were in agreement with these authors.

It is well-known that pork meat in general has an unacceptably high ratio of *n*-6 and *n*-3 polyunsaturated fatty acids [32].

The World Health Organization [33] recommended a reduction in the intake of SFA in favor of the n-3 polyunsaturated fatty acids (*n*-3 PUFA) for their beneficial effects on human health. Essentially, SFA are known to increase low-density lipoproteins and, in turn, the risk of cardiovascular disease. In our trial, despite that no significant increase in the concentration of ALA in EB pork meat was observed, the proportion of DPA was affected by the dietary treatment. The DPA is a long-chain *n*-3 PUFA that derives from ALA [34] thanks to the action of desaturase and elongase enzymes that catalyze the reactions; its beneficial effects on human health are well-known [35,36,37,38,39].

Furthermore, the longissimus thoracis et lumborum muscle from pigs fed with the EB diet tended to have a greater proportion of *n*-3 PUFA, while the proportion of n-6 PUFA was comparable between treatments and, consequently, the *n*-6/*n*-3 ratio was the lower in the EB treatment compared to control. A similar trend was observed by Scerra et al. [13] in meat by pigs fed a diet that included ensiled bergamot pulp supplement at the level of 15% dry matter.

The thrombogenic index (TI) was calculated to assess the potential for platelet aggregation. The TI tended to be reduced when the pigs were fed the EB diet compared with the control diet, and this can be explained by the fact that bergamot pulp affected some desirable fatty acids, such as those belonging to the *n*-3 family.

An accumulation of αlinolenic acid and long-chain fatty acids derived from it in the pig’s back fat is generally related to the levels of ALA in the diet provided. In this trial, exhausted bergamot by-product supplementation in the finishing diet of pigs significantly increased the concentration of ALA in the back fat. Consequently, the concentration of total *n-3* PUFA tended to increase in back fat from EB fed pigs. However, no differences were observed on long-chain *n-3* PUFA between the two groups.

Fatty acid composition determines the firmness/oiliness of adipose tissue and the oxidative stability of muscle, which, in turn, affects flavor and muscle color [40].

The Apulo-Calabrese breed is among those authorized for the production of the four protected-designation-of-origin cured meat products: “soppressata”, “salsiccia”, “pancetta” and “capocollo” of Calabria, all certified by the “Consortium for the protection of Calabria PDO cured meats”. In our study, we analyzed the salami, and we noticed that the use of exhausted bergamot pulp influenced, coherently with that observed in the meat and back fat, the fatty acid profile of the salami, enhancing some desirable fatty acids and improving indexes related to a lipid nutritional quality.

### 4.3. Oxidative Stability

Lipid oxidation is one of the main factors responsible for the loss of quality of meat and meat products. Following lipid oxidation, a series of unpleasant tastes and odors develop, as well as changes in color and texture. It is a rather complex process, where unsaturated fatty acids are involved and react with molecular oxygen to form peroxides, from which aldehydes, ketones and acids derive, many of which are responsible for the unpleasant rancid smell of oxidized fats [41].

Tocopherols are fat-soluble antioxidants and have a protective role against lipid oxidation [42]. Animals are unable to synthesize tocopherol, and its concentration in tissues is, therefore, strictly dependent on the diet. In the present study, although the concentration of vitamin E, especially α-tocopherol, in exhausted bergamot pulp was much higher than in the concentrate, no difference in vitamin E content was observed in meat of the two experimental groups. While for retinol, defined as a lipophilic scavenger, its concentration tended to be influenced by diet; in fact, it was greater in the meat of the EB group. As claimed by Halliwell and Gutteridge [43], in conditions of oxidative stress, there is a shift in the pro-oxidant/antioxidant balance in favor of the former in animal tissues. Despite the beneficial effects, *n*-3 PUFA are subject to oxidation during the processing and storage phases, inducing a potential alteration of the nutritional composition and product quality [44], and as the degree of unsaturation increases, the tendency toward oxidation also increases [45]. The results of the present study showed that the highly peroxidizable (HP)-PUFA ÷ Tocopherols ratio tended to increase (*p* = 0.057) in meat when exhausted bergamot pulp was integrated in the pig diet. It means that other possible compounds came into play, meaning that the use of exhausted bergamot pulp did not alter the shelf-life in raw meat. Agro-industrial by-products are rich in secondary compounds with antioxidant properties, such as phenolic compounds and essential oils, which, when used in animal nutrition, can affect the resistance of meat to oxidative deterioration. The peel of bergamot fruit contains a significant amount of flavonoids, in particular, naringin, neoeriocitrin and neohesperidin [46], compounds that have been found to have health-related properties, especially based on their antioxidant activity. Moreover, for their content of polyphenols and other bioactive phytochemicals, several agro-industrial by-products can be considered as functional feedstuffs [47]. The exhausted bergamot pulp used in our study showed a considerable residue of phenolic compound, as shown in Table 1 (>6 g TAe/kg DM). This supports the thesis that these compounds have come into play. Above all, phenolic compounds act against the oxidation of myoglobin by extending the shelf life of the product [48]. The discoloration of the meat is mainly due to the oxidation of myoglobin and the consequent accumulation of metmyoglobin [49]; this process causes the decrease in meat of redness (a*) and saturation (C*) values and the increase in hue angle (H*) during storage time. In the present study, the main descriptor of meat discoloration changed over the time of storage, as expected. Indeed, the meat yellowness (b*) and the hue angle (H*) increases during storage time. Dietary treatment significantly affected meat lightness (L*). This finding was consistent with the results from Priolo et al. [50] and Inserra et al. [51], who observed higher meat lightness in lambs and pork, respectively, fed with a diet containing carob pulp and therefore rich in tannins. Conversely, Crosswhite et al. [52] concluded that the inclusion of citrus pulp did not affect the main color descriptors.

In the study conducted by Luciano et al. [53], the authors reported that differences in oxidative stability between meat samples were evident with strong oxidative challenges, such as cooking, compared to samples stored in a refrigerated aerobic in darkness environment. In the present study, we also evaluated cooked meat by subjecting it to strong oxidative stress factors in order to highlight the influence of dietary treatment. Moreover, under these conditions, lipid oxidation (TBARS values) was not affected by the dietary treatment. Conversely, the dietary treatment significantly influenced the extent of lipid oxidation in salami, and the TBARS values were lower in the EB diet (Figure 1). The TBARS assay detects the level of malondialdehyde (MDA), which is the major lipid oxidation product [54]. The limit value that distinguishes the condition of rancidity is indicated in terms of MDA and is a maximum of 2 mg/kg [55]. In our study, in salami from the EB group, this value did not exceed this limiting threshold, while in salami from the control group, the level of MDA was already beyond of 2 mg/kg on the second day of monitoring. Furthermore, the TBARS values increased during aerobic refrigerated storage, and, after 5 days, the salami of the EB group showed lower values compared to the salami of the control group.

In salami, the concentration of the HP-PUFA increased when the pigs were fed the EB diet compared with the control diet, leading to an increase of the HP-PUFA ÷ Tocopherols ratio in salami from the EB group compared to the control group.

Despite the high levels of HP-PUFA, the TBARS values in the salami of animals fed with the EB were lower during the storage period, highlighting how salami from animals treated with bergamot responded better to the strong oxidative stresses, such as the grinding and the long storage time. The study of Luciano et al. [42] showed that vitamin E is the greatest contributor in the improvement of the antioxidant capacity of tissues. However, in our study in both raw meat and salami, no difference was found in the content of vitamin E between the two dietary treatments. Phenolic compounds may explain this result, as it was reported that dietary administration of hesperidin and naringin exerted a significant effect on the antioxidant capacity of broiler meat [56]. Therefore, it might be speculated that these phenolic compounds may have contributed to the delay in the lipid oxidation in salami.

Furthermore, Scerra et al. [13], in a study on salami, observed that by feeding pigs with a diet containing ensiled bergamot by-product, TBARS values were below the value of 2 mg MDA/Kg for the monitoring period. However, after 5 days of refrigerated storage, the TBARS values observed by Scerra et al. [13] in salami by feeding pigs with ensiled bergamot by-product were lower than the TBARS values observed in this trial by feeding pigs with a diet containing exhausted bergamot by-product. The highest amount of antioxidant compounds, such as phenols in the bergamot by-product used in the trial of Scerra et al. [13], could influence these differences (14.15 vs. 6.39 g TAe/kg DM, respectively, in ensiled bergamot by-product and exhausted bergamot by-product).

## 5. Conclusions

In view of these findings, the dietary inclusion of exhausted bergamot by-product in a diet of “Apulo-Calabrese” pigs could improve the fatty acids composition and oxidative stability of meat and salami. Indeed, the use of exhausted bergamot pulp in animal feeding, coming from a pharmaceutical industry after the extraction of phenolic compounds and then considered waste for the circular economy, has resulted in a qualitative improvement especially on the fatty acid profile of meat and meat products. The results of this study provided evidence that the use of the EB in pigs’ feeding can be practiced without negative effects on animal performance. Furthermore, this dietary strategy has significantly influenced the extent of lipid oxidation in salami. These results could be linked to the grinding process and the long storage time of the salami, resulting in a condition of strong oxidative stress, highlighting the best response from the meat of the animals of the group treated with dried bergamot pulp than the animals fed with only concentrate.

## Figures and Tables

**Table 1:** Chemical composition of experimental diets.

	Concentrate Diet	EB Diet	Exhausted Bergamot Pulp
Dry matter (DM) g/kg wet weight	881	892	937
Crude protein g/kg DM	132	131	133
Ether extract g/kg DM	31.6	31.3	30.4
Ash g/kg DM	40.2	40.9	50.9
NDF g/kg DM	439	437	508
Total phenolic compounds (g Tae ^1^/kg DM)	1.53	2.30	6.39
Tocopherols, μg/g dry matter			
α-Tocopherol	2.54	13.71	76.9
γ-Tocopherol	5.31	5.09	3.82
Fatty acids (g/100 g of total fatty acid)			
C14:0	0.12	0.37	1.81
C16:0	14.4	16.3	27.1
C16:1 *n-7*	0.15	0.49	2.38
C18:0	2.49	2.54	2.51
C18:1 *n-9*	31.5	27.6	7.67
C18:2 *n*-6	44.5	42.7	34.9
C18:3 *n*-3	2.11	4.33	16.9
C20:0	0.11	0.13	0.16

^1^ Tannic acid equivalent.

**Table 2:** Pigs’ performance in vivo and intake.

	Dietary Treatments ^1^		SEM ^7^	*p*-Value
	Control	EB		
Final BW ^2^, kg	151	153	2.356	0.243
Carcass weight, kg	127	123	5.132	0.679
Total DMI ^3^, g/d	3.46	3.53	0.147	0.321
ADG ^4^, g/d	465	481	8.001	0.472
FCR ^5^, g DMI ^3^/g ADG ^4^	7.44	7.33	0.229	0.191
Total FA ^6^ intake, g/d	41.3	53.8	2.331	0.004
C18:0 intake, g/d	1.03	1.34	0.119	0.073
C18:2 *n*-6 intake, g/d	18.4	22.1	0.341	0.098
C18:3 *n*-3 intake, g/d	0.87	3.88	0.117	0.001

^1^ Treatments were concentrate diet (control) and concentrate + 15% exhausted bergamot pulp (EB); ^2^ BW = body weight; ^3^ DMI = dry matter intake; ^4^ ADG = average daily gain; ^5^ FCR = feed conversion ratio; ^6^ FA = fatty acid; ^7^ SEM = standard error of means.

**Table 3:** Chemical composition of longissimus thoracis et lumborum muscle and salami (g/100 g wet weight).

	Dietary Treatments ^1^		SEM ^2^	*p*-Value
	Control	EB		
Chemical composition of LTL muscle				
Moisture	71.6	72.4	0.312	0.439
Crude protein	21.7	21.2	0.231	0.192
Ether extract	2.86	2.91	0.254	0.793
Ash	1.21	1.18	0.053	0.321
Chemical composition of salami				
Dry matter	71.5	72.9	0.623	0.187
Crude protein	29.1	28.8	0.421	0.395
Ether extract	17.2	16.1	0.874	0.481
Ash	6.7	6.4	0.191	0.443

^1^ Treatments were: concentrate diet (control) and concentrate + 15% exhausted bergamot pulp (EB). ^2^ SEM = standard error of means

**Table 4:** Effect of the dietary treatments on the oxidant vitamins (µg/g muscle) and fatty acid composition of longissimus thoracis et lumborum muscle (g/100 g of total fatty acids).

Item	Dietary Treatment		SEM ^4^	*p*-Value
	Control	EB		
Tocopherols and retinol, µg/g muscle				
α-Tocopherol	2.33	2.31	0.064	0.791
γ-Tocopherol	0.09	0.09	0.005	0.718
Retinol	7.11	9.9	0.797	0.078
Intramuscular fat, mg/100 g	2711	2942	191.4	0.345
C10:0	0.08	0.07	0.013	0.683
C12:0	0.10	0.08	0.009	0.234
C14:0	1.19	1.21	0.069	0.642
C14:1 *cis-9*	0.04	0.04	0.004	1.000
C15:0	0.04	0.04	0.006	0.891
C16:0	20.8	20.0	0.217	0.292
C17:0	0.42	0.41	0.031	0.632
C16:1 *cis-9*	3.33	3.39	0.101	0.716
C17:1 *cis-9*	0.20	0.22	0.009	0.293
C18:0	9.73	9.62	0.683	0.591
C18:1 *cis-9*	40.2	40.2	0.669	0.982
C18:1 *trans-11* (VA)	4.60	4.85	0.101	0.223
C18:2 *cis-9, cis-12* (LA)	11.8	11.4	0.573	0.432
C18:3 *n*-3 (ALA)	0.40	1.13	0.261	0.111
C20:0	0.27	0.22	0.027	0.213
C20:1 *cis-9*	0.86	0.79	0.057	0.322
C20:2 *n*-6	0.53	0.48	0.041	0.398
C20:3 *n*-3	0.20	0.44	0.076	0.131
C20:4 *n*-6	2.11	2.05	0.169	0.721
C20:5 *n*-3 (EPA)	0.10	0.18	0.028	0.144
C22:5 *n*-3 (DPA)	0.22	0.47	0.049	0.023
C22:6 *n*-3 (DHA)	0.20	0.32	0.046	0.123
∑ SFA ^1^	32.7	31.6	0.233	0.297
∑ MUFA ^1^	49.3	49.5	0.721	0.811
∑ PUFA ^1^	15.6	16.5	0.672	0.319
∑ *n*-3 PUFA	1.12	2.54	0.321	0.080
∑ *n*-6 PUFA	14.5	13.9	0.632	0.298
*n*-6/*n*-3 PUFA	12.9	5.48	0.901	0.009
∑ PUFA ^1^/∑ SFA ^1^	0.48	0.52	0.021	0.193
Thrombogenic index	0.90	0.78	0.024	0.064
Atherogenic Index	0.40	0.38	0.008	0.135
HP-PUFA ^2^ (mg/g muscle)	0.64	1.00	0.121	0.110
HP-PUFA ÷ Tocopherols ^3^	2.43	2.57	0.051	0.057

^1^ SFA, saturated fatty acids; MUFA, monounsaturated fatty acids; PUFA, polyunsaturated fatty acids. ^2^ HP-PUFA, highly peroxidizable PUFA; ^3^ expressed as mg/g muscle; ^4^ SEM = standard error of means.

**Table 5:** Effect of the exhausted bergamot by-product on back-fat fatty acid profile.

Item (g/100 g of total Fatty Acids)	Dietary Treatment		SEM ^1^	*p*-Values
	Control	EB		
C10:0	0.06	0.06	0.012	0.892
C12:0	0.14	0.12	0.007	0.321
C14:0	1.45	1.43	0.039	0.221
C14:1 cis-9	0.03	0.04	0.007	0.895
C15:0	0.09	0.07	0.009	0.150
C16:0	21.3	20.1	0.232	0.211
C17:0	0.48	0.47	0.019	0.355
C16:1 cis-9	2.12	2.03	0.042	0.544
C17:1 cis-9	0.28	0.36	0.038	0.036
C18:0	9.19	9.81	0.643	0.353
C18:1 cis-9	42.8	40.4	0.684	0.112
C18:1 trans-11 (VA)	2.89	3.01	0.066	0.289
C18:2 cis-9, cis-12 (LA)	14.9	15.1	0.326	0.332
C18:3 *n*-3 (ALA)	0.52	1.21	0.142	0.037
C20:0	0.27	0.25	0.008	0.812
C20:1 cis-9	1.17	1.28	0.059	0.156
C20:2 *n*-6	0.68	0.65	0.102	0.823
C20:3 *n*-3	0.13	0.19	0.039	0.245
C20:4 *n*-6	0.21	0.16	0.018	0.345
C20:5 *n*-3 (EPA)	0.19	0.19	0.018	0.856
C22:5 *n*-3 (DPA)	0.19	0.26	0.029	0.133
C22:6 *n*-3 (DHA)	0.17	0.24	0.049	0.443
∑ SFA ^2^	33.0	32.3	0.765	0.433
∑ MUFA ^2^	49.3	47.1	0.755	0.212
∑ PUFA ^2^	17.0	18.0	0.412	0.072
∑ *n*-3 PUFA	1.21	2.09	0.202	0.057
∑ *n*-6 PUFA	15.8	15.9	0.414	0.377
*n*-6/*n*-3 PUFA	13.1	7.63	1.263	0.093
∑ PUFA/∑ SFA	0.52	0.56	0.061	0.234

^1^ SEM = standard error of means; ^2^ SFA, saturated fatty acids; MUFA, monounsaturated fatty acids; PUFA, polyunsaturated fatty acids.

**Table 6:** Effect of exhausted bergamot by-product on the antioxidant vitamins and fatty acid profile of salami (g/100 g of total fatty acids).

Item	Dietary Treatment		SEM ^4^	*p*-Values
	Control	EB		
Tocopherols and retinol, µg/g salami				
α-Tocopherol	0.55	0.59	0.432	0.792
γ-Tocopherol	0.05	0.05	0.009	0.321
Retinol	3.26	3.11	0.521	0.321
Total fat, g/100 g of salami	19.6	16.8	47.54	0.099
C12:0	0.14	0.17	0.022	0.321
C14:0	1.48	1.31	0.069	0.098
C16:0	22.0	20.2	0.542	0.099
C16:1 *cis-9*	2.29	2.36	0.139	0.321
C18:0	10.9	9.51	0.357	0.036
C18:1 *cis-9*	45.0	43.8	0.445	0.161
C18:2 *cis-9, cis-12* (LA)	11.1	11.9	0.221	0.081
C18:3 n-3 (ALA)	0.46	2.08	0.239	<0.001
C20:1 *cis*-9	1.11	1.12	0.039	0.234
C20:2 *n*-6	0.56	0.60	0.057	0.387
C20:4 *n*-6	0.32	0.29	0.074	0.543
C20:5 *n*-3 (EPA)	0.19	0.26	0.043	0.432
C22:5 *n*-3 (DPA)	0.19	0.22	0.041	0.456
C22:6 *n*-3 (DHA)	0.14	0.17	0.032	0.298
∑ SFA ^1^	34.6	31.2	0.834	0.039
∑ MUFA ^1^	48.4	47.3	0.536	0.365
∑ PUFA ^1^	12.9	15.5	0.443	<0.001
∑ *n*-3 PUFA	0.98	2.73	0.256	<0.001
∑ *n*-6 PUFA	11.9	12.8	0.258	0.065
*n*-6/*n*-3 PUFA	12.2	4.7	1.378	<0.001
∑ PUFA ^1^/∑ SFA ^1^	0.37	0.49	0.033	0.004
Thrombogenic index	1.03	0.80	0.049	0.001
Atherogenic Index	0.46	0.41	0.017	0.038
HP-PUFA ^2^ (mg/g salami)	1.20	3.51	0.345	0.001
HP-PUFA ÷ Tocopherols ^3^	3.20	3.76	0.111	0.033

^1^ SFA, saturated fatty acids; MUFA, monounsaturated fatty acids; PUFA, polyunsaturated fatty acids. ^2^ HP-PUFA, highly peroxidizable PUFA; ^3^ expressed as mg/g muscle; ^4^ SEM = standard error of means.

**Table 7:** Effect of the dietary treatment and time of refrigerated storage on raw meat color stability and lipid oxidation of raw and cooked meat.

	Dietary Treatment ^1^		Time (T) ^3^			SEM	*p*-Values	Time	D×T
	Control	EB	0	1	2		Diet		
L* values ^2^	41.9	43.9	42.4	43.5	43.4	0.562	0.038	0.501	0.932
a* values ^2^	6.3	6.4	6.7	6.4	5.9	0.343	0.421	0.178	0.132
b* values ^2^	8.2	9.3	6.9 ^x^	9.9 ^y^	8.7 ^z,y^	0.321	0.060	0.007	0.085
C* values ^2^	10.6	11.3	9.9	11.7	10.9	0.332	0.143	0.079	0.059
H* values ^2^	53.6	55.4	42.4 ^x^	57.0 ^y^	59.1 ^y^	1.43	0.453	0.001	0.754
TBARS raw meat, mg MDA/kg	0.52	0.55	0.47	0.50	0.51	0.017	0.457	0.293	0.377
TBARS cooked meat, mg MDA/kg	2.96	2.70	2.02 ^x^	2.68 ^y^	3.79 ^z^	0.175	0.165	0.001	0.467

^x,y,z^ Within row, different superscript letters indicate differences (*p* < 0.05) between days of storage tested by using the Tukey’s adjustment for multiple comparisons. ^1^ Treatments were concentrate diet (control) and concentrate + 15 % exhausted bergamot pulp (EB); ^2^ L* = lightness; a* = redness; b* = yellowness; C* = Chrome; H* = hue angle, measured in degrees; ^3^ Times, 0, 1, 2 = days 0, 3, 7 (raw meat slices); days 0, 2, 5 (cooked meat slices).

## Data Availability

The data presented in this study are available on request from the corresponding author.
